# Hydroxyproline Ring Pucker Causes Frustration of Helix Parameters in the Collagen Triple Helix

**DOI:** 10.1038/srep12556

**Published:** 2015-07-29

**Authors:** W. Ying Chow, Dominique Bihan, Chris J. Forman, David A. Slatter, David G. Reid, David J. Wales, Richard W. Farndale, Melinda J. Duer

**Affiliations:** 1 Department of Chemistry, University of Cambridge, Lensfield Road, Cambridge CB2 1EW, UK; 2 Department of Biochemistry, University of Cambridge, Downing Site, Cambridge CB2 1QW, UK; 3 Institute of Infection and Immunity, School of Medicine, Cardiff University, Cardiff CF14 4XN, UK

## Abstract

Collagens, the most abundant proteins in mammals, are defined by their triple-helical structures and distinctive Gly-Xaa-Yaa repeating sequence, where Xaa is often proline and Yaa, hydroxyproline (Hyp/O). It is known that hydroxyproline in the Yaa position stabilises the triple helix, and that lack of proline hydroxylation *in vivo* leads to dysfunctional collagen extracellular matrix assembly, due to a range of factors such as a change in hydration properties. In addition, we note that in model peptides, when Yaa is unmodified proline, the Xaa proline has a strong propensity to adopt an endo ring conformation, whilst when Yaa is hydroxyproline, the Xaa proline adopts a range of endo and exo conformations. Here we use a combination of solid-state NMR spectroscopy and potential energy landscape modelling of synthetic triple-helical collagen peptides to understand this effect. We show that hydroxylation of the Yaa proline causes the Xaa proline ring conformation to become metastable, which in turn confers flexibility on the triple helix.

Fibrillar collagens are the dominant proteins in mammalian tissue extracellular matrix. Although often viewed as static, solid proteins, extracellular matrix collagens were shown some 30 years ago to exhibit significant dynamical fluctuations^1^, and more recent studies have shown that model collagen-like peptides, at least, undergo structural changes upon binding proteins^2^,^3^,^4^, strongly suggesting that if replicated in native collagens, collagen molecular flexibility is of biological, as well as mechanical, importance. However, the corresponding molecular features that underlie the flexibility of collagens is not understood.

At a molecular level, collagens are triple-helical proteins with a repetitive Gly-Xaa-Yaa primary structure in each of the three constituent chains. Glycine is frequently followed by proline and hydroxyproline in the Xaa and Yaa positions, respectively. A key point here is that hydroxyproline (O or Hyp), an ascorbic acid–dependent post-translational modification of proline, is absolutely necessary for maintaining the stability of native collagens and severe pathologies arise when the post-translational modification of proline to hydroxyproline is disrupted, manifest for instance by scurvy in ascorbic acid deficiency.

GPO sequences constitute around 10% of all Gly-Xaa-Yaa triplets in native collagens^5^. Synthetic peptides containing sufficient (GPO)_n_ and (GPP)_n_ can self-assemble in water to give a collagen-like triple helix. These structures have helix parameters close to 7/2 helical symmetry (20.0 Å axial repeat, synonymous with 7_5_)^6^,^7^,^8^,^9^, while fibre X-ray diffraction of native tendon collagen has indicated a looser 10/3 helical symmetry (28.6 Å axial repeat, synonymous with 10_7_) in collagen proteins *in vivo*^10^. The difference in helical parameters between imino-rich model peptides and native collagens is generally ascribed to the native collagens having significant amino-rich segments, presumably adopting looser helical structures, in addition to imino-rich parts.

The backbone geometry of the imino residues, and hence the molecular helical geometry, is strongly coupled to the prolyl ring conformation, which gives the intriguing possibility that the molecular helical geometry can be in large part be dictated by the steric and electronic factors that determine the ring conformation. Hence, the conformation of prolyl rings in (GPO)_n_ and (GPP)_n_ is a well-studied feature of triple helical peptides, with the expectation that general principles in these model systems will be replicated in native collagen triple helices. In general, imino acids in the Yaa position (denoted henceforth as P_Y_ and O_Y_ for proline and hydroxyproline respectively), tend to take the exo conformation (proline C_γ_ puckered towards the opposite side of the pyrrolidine ring to the exocyclic C–CO bond, also known as “up”), whilst proline in the Xaa position (P_X_) adopts an endo conformation (“down”)^6^,^7^,^11^. Experiments^11^,^12^ and calculations^13^ show clearly that the exo conformation of prolyl rings in the Yaa position stabilises the collagen triple helix, hence the fact Yaa prolines in native collagenous tissues are predominantly modified to exo-preferring hydroxyproline (exo-preferring through a combination of a gauche effect and an *n* → π^*^ interaction)^14^,^15^,^16^.

The gain in thermodynamic stability from having prolyl rings in the exo conformation at the Yaa position in collagen-like peptides can be readily understood in structural terms—the exo conformation gives a backbone structure at the Yaa position that closely aligns with an ideal collagen 7/2 triple helical structure. Exo Yaa residues thus preorganise the peptide chain closer to the structure required in the 7/2 triple helix, and thus triple helix formation comes at a lower entropic cost than for chains where the Yaa position conformation is not preorganised^12^. A similar situation exists for the endo conformation of proline residues in the Xaa position—rings in the endo conformation have a backbone geometry that closely aligns with the ideal 7/2 triple helical geometry, just as exo conformations in the Yaa position do. Model collagen peptides incorporating non-native proline derivatives in the Xaa positions that strongly favour endo conformations lead to very significantly enhanced stability of the collagen triple helix, as demonstrated by the much higher melting temperatures of such peptides, i.e. the temperature at which the three chains of the triple helix dissociate in aqueous solution, compared to a (GPP)_n_ peptide of the same length^17^,^18^,^19^. This then brings us to the intriguing point: although GPP-based model collagen peptides show that P_X_ rings in GPP triplets strongly favour endo conformations (as assessed by XRD analyses), the P_X_ rings in GPO-based model collagen peptides, i.e. in native-like sequences, do *not* in fact have a strong preference for the endo conformation. So whilst the P_X_ rings in triple helices containing only GPP sequences have almost exclusively endo conformations (e.g. PDB 3AH9 by Okuyama *et al.*), in (GPO)_9_, for instance, the P_X_ rings occupy endo and exo conformations nearly equally. For example, of all the P_X_ rings that were determined in the PDB structure 3B0S^20^, 29 endo and 24 exo ring conformations were observed. The clear question here is why do P_X_ rings in GPO sequences apparently not favour a geometry that fits with the expected collagen 7/2 triple helix backbone structure, and which would confer further thermodynamic stability? Thermodynamic stability is a feature which one might intuitively expect to be advantageous for a structural protein, especially one with a long half-life as the majority of collagens have in mammals. This is potentially a very important question—the difference in the prolyl backbone *ϕ* dihedral angle between endo and exo conformations is typically ≈15° and can be as much as 45°^21^. In other words, deviation of the P_X_ rings from the endo conformation in GPO sequences potentially represents a very significant distortion from the ideal 7/2 triple helical structure, the very structure that the incorporation of hydroxyproline into the triplet was evolved to maintain. This apparent paradox strongly suggests that there are biological requirements for the collagen molecular structure that is beyond simply thermodynamic and structural stability. The further question then is, what is the biological relevance of the conformational promiscuity of P_X_ rings in GPO triplets?

NMR spectroscopy has shown that a dynamic equilibrium exists between endo and exo states of prolyl rings in collagen, model collagen peptides, and other related proteins^1^,^22^,^23^ which in turn will affect the average collagen molecular geometry observed on timescales slower than the ring flipping process. In (solid) native collagen, the molecular motions of the proline C_γ_ atom samples angular displacements of 20°–30°, and hydroxyproline C_γ_ 10°–18°, on timescales of 10 μs to sub-nanoseconds, dynamic processes most likely corresponding to proline ring flipping^1^. These timescales are likely to be significantly faster than protein binding processes, and so the average collagen molecular geometry presented to proteins will depend on the relative populations of the endo and exo states of the proline and hydroxyproline rings.

Here we show that when hydroxyproline is in the Yaa position of a collagen triple helix, Xaa proline rings have a frustrated geometry and flip rapidly between near-equally populated endo and exo conformations. This confers considerable flexibility on the peptide triple helix as a whole whilst maintaining the overall structure within clear boundaries. In contrast, Xaa prolines adjacent to Yaa unmodified prolines are more likely to be found “anchored” in the relatively deep potential energy minimum of the endo conformation, giving a less flexible structure.

Our approach is to determine Xaa proline ring endo:exo conformational populations experimentally using ^13^C NMR chemical shift measurements, combined with a potential energy landscape analysis^24^ to understand details of the ring-flipping dynamics at biologically-relevant temperatures. Our conclusion is that the rapid Xaa proline ring flipping, inherent in GPO triplets, results in an overall molecular geometry that flexes between 7/2 and tighter helical structures at biologically-relevant temperatures. This result implies that hydroxylation of Yaa prolines in collagens is not only *essential* for triple helix stability—it confers well-defined molecular *flexibility* at neighbouring Xaa prolines, which may have considerable biological significance and which may be critical in explaining pathologies associated with lack of Yaa proline hydroxylation.

## Results

Our primary aim is to understand the effect of hydroxylation of Yaa position prolines on the conformation and structure of the preceding Xaa-position proline, i.e. we want to compare and understand the relative ring conformer populations for P_X_ in GPP versus GPO triplets. Whilst the distribution of proline ring conformations found from XRD structural analysis of model collagen peptides can be expected to broadly reflect the conformational propensity of proline rings, limits in resolution mean that XRD structural models cannot be expected to reproduce accurate ring conformation populations. Moreover, XRD data is often collected at 100 K, at which the ring conformation populations are likely to be very different from those at biologically-relevant temperatures.

Our protocol for experimentally determining the endo:exo populations of proline ring conformations uses the fact that ^13^C NMR shifts are sensitive to the ring conformation of imino residues. The rapid rate of ring flipping at all temperatures of biological interest means that the ^13^C chemical shifts observed are the equilibrium population–weighted average of those for the respective endo and exo conformations. Thus, proline ring ^13^C isotropic shifts are a direct measure of endo:exo populations at any temperature of interest. Proline C_γ_^13^C chemical shifts in particular are accurate sensors of proline ring conformation^25^, being sensitive to the dihedral angles describing the local bond geometry around this carbon. In type II poly-L-proline, a chemical shift difference of 1.5 ppm is observed between the (half chair) endo and exo conformations^26^, the endo conformation giving a C_γ_^13^C chemical shift of 23.8 ppm and exo, 25.5 ppm. In the collagen model peptides of this study, we expect C_γ_ chemical shifts approximately in this range, the precise value being determined by the endo:exo ratio for the imino residue ring being observed in the NMR spectrum, i.e. we will measure chemical shift values of *n*_endo_·*δ*_endo_+*n*_exo_·*δ*_exo_ where *n*_endo_ and *n*_exo_ are the fractions of rings in the endo and exo conformational states respectively, and *δ*_endo_ and *δ*_exo_ are the isotropic ^13^C shifts of the pure endo and exo conformations.

Two model peptides were synthesised, (GPO)_11_ and (GPO)_5_GPP(GPO)_5_, in which specific residues of the central triplet in each case, i.e. GPO and GPP respectively, were U-^13^C,^15^N-labelled in each chain of the homotrimeric triple helices (Table 1). Placing a GPP triplet in an otherwise GPO oligomer allows us to compare the local structure of GPO and GPP triplets within otherwise near identical molecules. Additionally, we synthesised (GPP)_11_ with the central triplet P_Y_ U-^13^C labelled for structural comparison with the (GPO)_5_GPP(GPO)_5_ GPP triplet, as there have been XRD structural analyses of pure (GPP)_n_ peptides, but not GPP substituted into (GPO)_n_ oligomers. The isotope labelling enables us to resolve NMR signals from specific residues in the central Gly-Xaa-Yaa triplet in each case, thus avoiding overlap of signals from all proline sites and so maximizing the sensitivity of ^13^C chemical shifts to proline ring conformation^25^,^26^,^27^. The nomenclature assigned to these variously labelled peptides is summarised in Table 1; **GPO-GX**, for example, refers to (GPO)_11_, i.e. dominant NMR signals are from a GPO triplet, with U-^13^C,^15^N-labeled Gly and P_X_. We use lyophilised samples so that the NMR spectra contain signals from a representative collection of possible structures for each peptide, rather than a single structure, and thus probe a range of accessible prolyl ring conformations.

**Figure 1:**
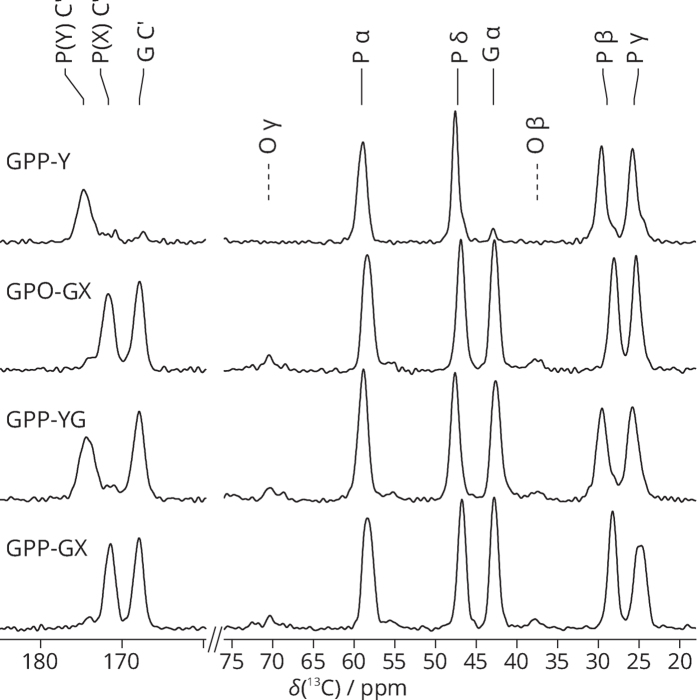
^13^C CP-MAS ssNMR spectra of triple-helical peptides used in this study to model GPO and GPP triplets in the collagen triple helix.

^13^C NMR spectra of the peptides are, as expected, dominated by signals from the labelled residues (Fig. 1), and thus ^13^C chemical shift assignment for the model peptides is trivial, although line widths and low spectral dispersion preclude sub-assignment of signals from specific atoms to leading, middle and trailing peptide strands of the triple helices. In comparing ^13^C and ^15^N chemical shifts between these peptides, we are mindful of the fact that Pro/Hyp exert a particularly strong effect on the N, CO, C_α_ and C_β_ chemical shifts of the *i* − 1 (preceding) residue in a peptide chain, which is the so-called “proline effect”^28^,^29^ That this effect does not extend to the C_γ_ and C_δ_ chemical shifts is evidenced from substantial literature chemical shift data^29^.

### Proline Ring Conformation and Chemical Shift Trends

Based on XRD results^7^,^30^,the ^13^C ,^15^N-labeled P_Y_ in both **GPP11-Y** and **GPP-YG** are expected to favour exo conformations. Thus we assign the chemical shifts for P_Y_ in these two peptides as being representative of a population that is biased towards a pure exo conformational state. The similarity of conformation for the labelled proline in these two peptides is clear from the very similar ^13^C chemical shifts throughout the P_Y_ residues.

Proline C_γ_^13^C chemical shifts for endo conformations are expected to be lower than those for exo cf. poly-L-proline (Table 1). That this is indeed the case is confirmed by comparing the C_γ_^13^C chemical shifts for exo-favouring P_Y_ in the GPP triplets of **GPP11-Y** and **GPP-YG** (both 25.7 ppm) with those for P_X_ in the GPP triplet of **GPP-GX**—expected to strongly favour endo. The P_X_C_γ_ for the GPP triplets in fact give two ^13^C signals, chemical shifts 24.4 and 25.0 ppm, both significantly lower frequency than for the largely exo conformation P_Y_ rings, as expected by analogy with poly-L-proline.

**Figure 2:**
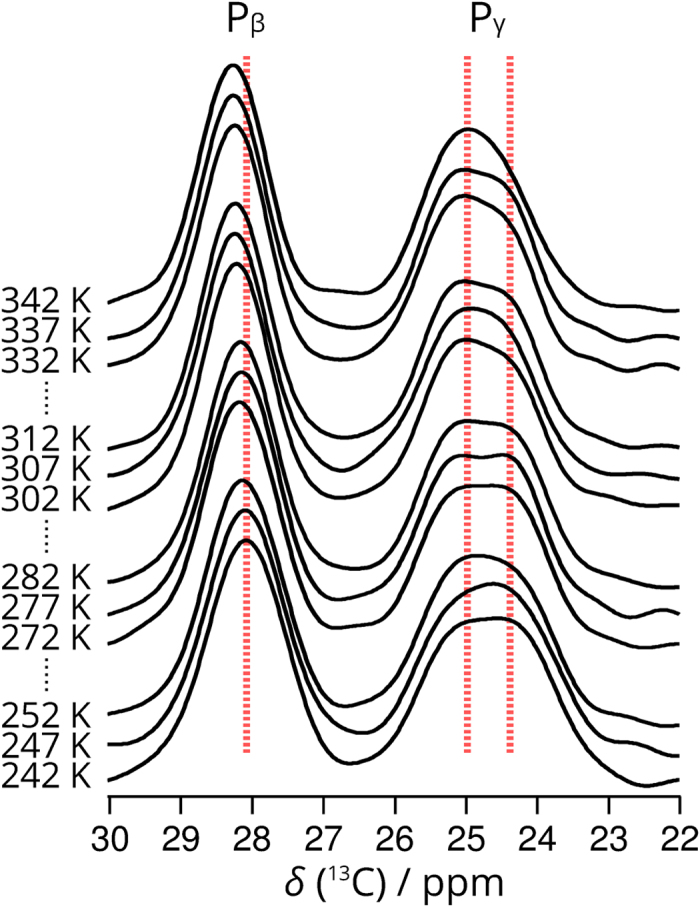
C_β_ and C_γ_ signals from variable-temperature ^13^C CP-MAS ssNMR spectra for **GPP-GX** at 242–342 K. Dashed lines are aligned with signal position at the lowest temperature, highlighting the existence of two C_γ_ signals over a wide temperature range. Signal maxima are tabulated in Supplementary Information, Tables S2–S4. NMR spectra for all signals at all temperatures are shown in Figure S1–S2.

**Figure 3:**
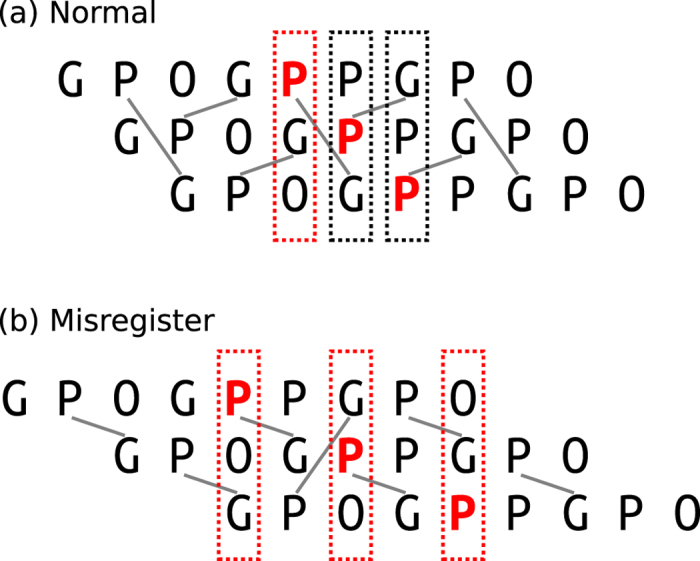
Schematic diagram of the chain register for triple-helical **GPP-GX**. The ^13^C-labeled proline is highlighted in red, and grey lines indicate inter-chain hydrogen bonds. (**a**) Normal register with a one residue stagger between chains in the triple helix. (**b**) One possible misalignment of the chains with a stagger of two residues, causing misregister. Rectangles indicate the two possible helix cross sections: GPP (black dots) or GPO (red dots).

That there are two signals for the GPP P_X_C_γ_ in **GPP-GX** over a wide range of temperature (Figure 2) implies there are two distinct endo:exo population distributions for the GPP triplet P_X_ residue in this molecule. That two C_γ_^13^C signals only occur for the peptides in which a GPP triplet is substituted within an otherwise GPO oligomer suggests that the source of the two signals in this case is the GPP P_X_ residue having two different possible environments created by the neighbouring chains in the triple-helix structure. Figure 3(a) shows that this is indeed the case. The chains of the triple helix are normally shifted by one residue with respect to each neighbouring chain as shown in this figure, the so-called register of the three peptide chains. This means that in any cross section of the triple helix, the three residues in the cross section will consist of a glycine, an Xaa (here P_X_) residue and a Yaa residue. For the cross sections containing the ^13^C-labelled GPP P_X_ residues (i.e. one P_X_ ring in each chain), the Yaa residue will be proline in two cross sections and hydroxyproline in one cross section. Thus, there are two different cross sections possible for the three GPP triplets in the molecule and so we expect two C_γ_ signals for the GPP P_X_ residues, one associated with the G(^13^C-P)P cross sections and one associated with the G(^13^C-P)O cross sections. The signal at 25.0 ppm coincides closely in frequency with that for the P_X_ C_γ_ signal for the (GPO)_11_ molecule (25.3 ppm in the latter), a molecule which consists entirely of GPO cross sections, and thus we can confidently assign the 25.0 ppm signal for the (GPO)_5_GPP(GPO)_5_ P_X_ C_γ_ to be from P_X_ C_γ_ in GPO cross sections, and hence the signal at 24.4 ppm, we assign to the P_X_ C_γ_ in GPP cross sections. If all the molecules of the sample are in perfect register, then we would expect an intensity ratio of 2:1 for these two signals. However, chain misregisters (Fig. 3(b)), i.e. chain registers with the three peptide chains offset by two or more residues are highly likely. Key here is that (GPO)_n_ triple helices are significantly less stable than (GPO)_n_ triple helices, as evidenced by the much lower melting temperatures for (GPO)_n_ triple helices. When the three chains are in perfect register, the molecule contains a clear section of GPP helix—presumably a less stable section than the sections containing only GPO triplets—which is dispersed by the chains misregistering. Thus, we can expect an equilibrium of chain registers in the sample. In any misregistered molecule, the three ^13^C-labelled P_X_ rings are all in GPO cross sections, and all three P_X_C_γ_ sites would thus be expected to give signals at 25.0 ppm. We observed that the two GPP P_X_ C_γ_ signals have approximately equal intensities at 242 K (see Fig. 1) which suggests that some of the (GPO)_5_GPP(GPO)_5_ molecules are in some form of misregister at this temperature.

The P_X_C_γ_ chemical shift associated with the GPP cross section, 24.4 ppm, is close to that expected for a pure endo population of conformations and so implies that the P_X_ rings in this cross section at least strongly favour the endo conformation, in line with conclusions from XRD analyses of GPP oligomers. In turn, the higher P_X_C_γ_ chemical shift of 25.0 ppm for the P_X_ in GPO cross sections implies a P_X_ ring conformation with rather more equal endo and exo conformations in this case. The shift towards a larger exo population is even more pronounced for the P_X_ ring in the GPO sequence (which necessarily also has a GPO cross section) in (GPO)_11_, where the C_γ_ chemical shift is 25.3 ppm, i.e. intermediate between the chemical shifts expected for endo and exo conformations.

The key question then is: why does hydroxylation at the Yaa position within a GXY triplet or a helix cross section appear to affect the P_X_ ring conformational population distribution so markedly? Specifically, why does P_X_ in a GPO triplet appear to have a much less strong preference for either endo or exo conformations than P_X_ in GPP triplets in GPP cross sections? It is known that the proline (and presumably hydroxyproline) ϕ backbone dihedral angle is coupled to the ring conformation in peptides in general. This implies that when Yaa positions are occupied by strongly exo-favouring hydroxyproline, the time-averaged backbone ϕ dihedral angle for the Xaa residue is different from that when Yaa is occupied by less strongly exo-favouring, unmodified proline. Since the 7/2 helical geometry is most closely matched by P_X_ in an endo conformation, the further implication is that the more equal endo:exo populations for P_X_ in GPO triplets confers a P_X_ backbone geometry that deviates significantly from the ideal for a 7/2 triple helix. The next question is then what is the helical geometry at the P_X_ site in this case? Answering these questions will give us insight into the possible biological relevance of the conformational promiscuity of the P_X_ ring conformation in GPO sequences.

We can answer these questions by modelling the potential energy landscape available to the proline rings in these triple helical molecules. Specifically, we can quantify changes in the landscape introduced by hydroxylating Y-position prolines in a collagen triple helix, and so understand why P_X_ in a GPO triplet behaves so differently from a GPP triplet in terms of its conformational preference.

### Analysis of the Potential Energy Landscape

There have been many excellent studies modelling the factors that affect the stability of the collagen triple helix and collagen model peptides^11^, and modelling the dynamics between proline and hydroxyproline ring conformational states^31^,^32^,^33^,^34^,^35^. These have largely focused on single or small numbers of residues within a peptide. In our work, we need access to equilibrium ring conformation populations so that we can compare with experimental data, and so we need to model the potential energy landscape in molecules in which there are many P_X_ rings in similar environments. We therefore modelled the potential energy landscape for imino residue ring flips for the entire molecule in (POG)_12_ and (PPG)_12_, so that we can compare the P_X_ ring conformation populations between GPP triplets in purely GPP cross sections and GPO triplets in purely GPO cross sections.

**Figure 4:**
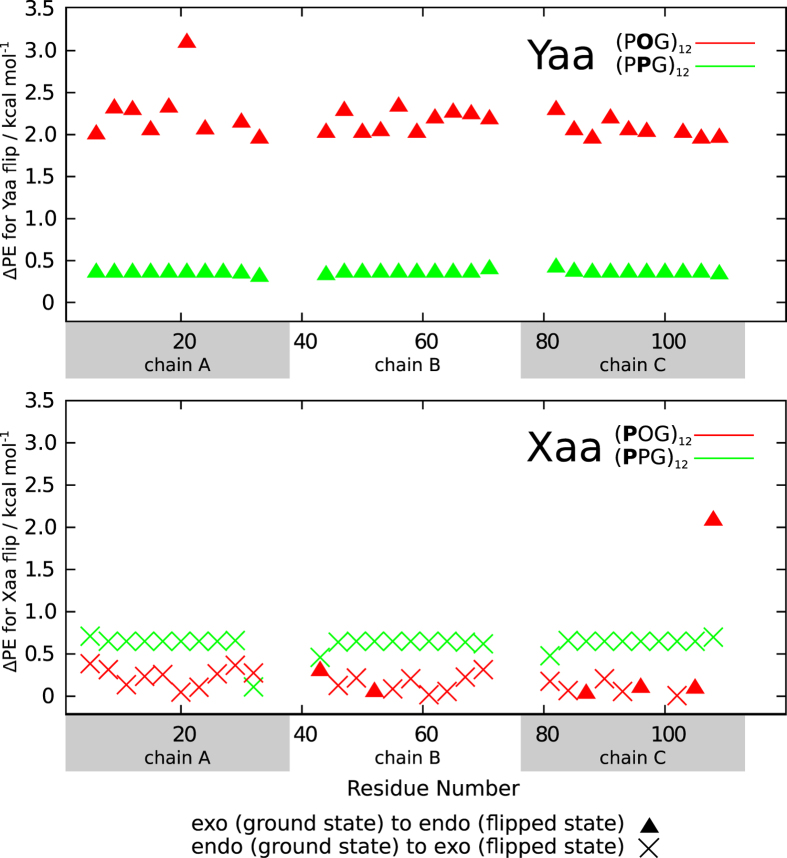
Modelling the potential energy change resulting from a single flip of each proline or hydroxyproline ring in (POG)_12_ and (PPG)_12_. Top panel: Yaa rings; bottom panel: Xaa rings. The potential energy changes for the terminal rings are not shown. All points were plotted relative to their respective ground state potential energy which is taken to be energy zero.

The results of these calculations are summarised in Fig. 4, which shows the potential energy change as each proline ring in the ground state conformation of (POG)_12_ and (PPG)_12_ is independently flipped to the opposite conformational state.

As expected, P_Y_ in (PPG)_12_ and O_Y_ in (POG)_12_ adopt predominantly exo states, with the preference stronger for hydroxyproline, for reasons which are already well-established^11^. P_X_, for the most part, adopts endo conformations in the ground state of both peptides. The key finding here is that the potential energy change for P_X_ ring flip in (POG)_12_ is about half of that for P_X_ in (PPG)_12_. The presence of the OH group on hydroxyprolines in the Yaa position is the only difference between these two molecules, and so it must account for the reduced potential energy difference between the ring conformations in the Xaa position, in addition to the known increased potential energy difference between exo and endo conformations in the Yaa position for hydroxyproline compared with proline, and the resulting higher energy barriers for the hydroxyproline ring flips in the Yaa position, i.e. slower ring flips for hydroxyproline. Also of note is that, for all rings, the potential energy differences between endo and exo conformations are small compared to the thermal energy (*kT*) for temperatures above around 200 K. This is in agreement with the NMR studies^1^,^22^,^23^,^36^, which have shown that proline rings in collagen-like peptides are not in a single conformational state, but are flipping rapidly between endo and exo states. Further analysis (see Supplementary Information, Section S3) shows that there is little coupling between rings, and so the difference in potential energy between different ways of flipping two rings of a given type, i.e. two P_X_ or one P_X_ and one P_Y_/O_Y_ etc. is largely independent of where the rings are in the peptide.

**Figure 5:**
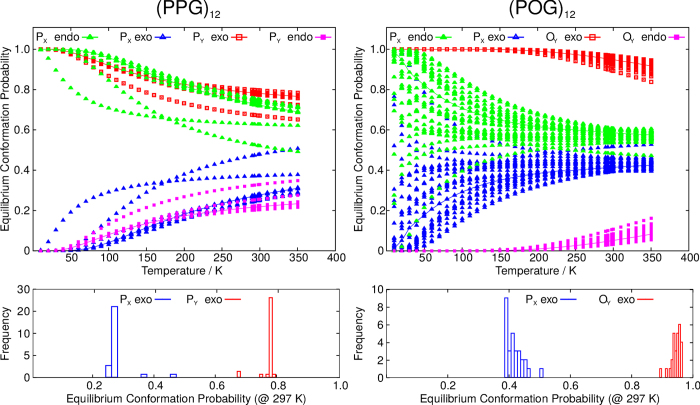
Top: Calculated distributions of ring conformations as a function of temperature for (PPG)_12_ (left) and (POG)_12_ (right). Bottom: Histograms showing the distributions of ring conformation populations at 297 K. Note the difference in vertical scaling.

We can calculate the relative populations of the endo:exo conformations as a function of temperature (Fig. 5) using information from the potential energy landscape (harmonic superposition approximation)^24^. For (POG)_12_ at room temperature, there is a broad distribution of both P_X_ and O_Y_ ring conformation populations, centred at 60:40 endo:exo for P_X_, and 5:95 endo:exo for O_Y_, which is consistent with our NMR observations, and with X-ray diffraction (XRD) structures for analogous crystalline POG oligomers^20^. The behaviour for (PPG)_12_ is very different from (POG)_12_. For P_Y_, the distribution is centred at 22:78 endo:exo, a significantly less strong preference for exo than P_Y_ has in (POG)_12_, which is consistent with expectations from theoretical analysis^31^,^32^,^33^,^34^,^35^ and XRD. For P_X_ in (PPG)_12_, the relative population of ring conformations is very tightly centred on 72:28 endo:exo—only two P_X_ rings close to the ends of the molecule have endo:exo ratios significantly different from this. Again, this is consistent with the distribution of endo:exo conformations for (PPG)_10_ found from XRD structures^7^,^30^ and our measured C_γ_^13^C NMR chemical shifts. Indeed, we can use these calculated ring conformational distributions with our experimental ^13^C chemical shift data to calculate the expected ^13^C chemical shifts for P_X_ pure endo and exo states (for GPP and GPO triplets) as 23.7 ppm and 26.3 ppm respectively.

These results suggest that stabilization of the Yaa exo state in GPO triplets, and the corresponding strong preference for this conformation at the Yaa position, leaves the neighbouring P_X_ ring in a metastable state, with the P_X_ ring moving rapidly over nearly the entire conformational range. Thus hydroxylation of the Yaa-position proline not only stabilises the collagen triple helical structure, it confers considerable flexibility at the Xaa-position proline.

The next question is: does this flexibility of the P_X_ residue have any significant effect on the backbone structure of these collagen model peptides? In general, endo—exo proline ring flips in proteins can change the proline ring dihedral angle by around 10° or more. Table 2 details the calculated changes in the peptide backbone torsion angles (ϕ and ψ) that arise when changing the proline ring conformation for Xaa and Yaa prolines, and shows that there are differences of around 11° or more in P_X_ backbone ϕ dihedral angle between endo and exo ring conformations.

Another pertinent question at this point is: how do these local structural changes arising from ring flipping affect the time-averaged overall molecular geometry at biologically-relevant temperatures? The geometric structures that correspond to minima in the potential energy landscape may be encountered by the molecule at any temperature. We computed two such structures with different imino residue ring conformations. The first, the *ground state* (lowest potential energy) geometric structure for (POG)_12_ has P_X_ endo (very largely) and O_Y_ exo. In the second structure 40% of the P_X_ rings were selected at random and flipped to the exo state, representing one possible configuration of the P_X_ exo rings expected around room temperature.

**Figure 6:**
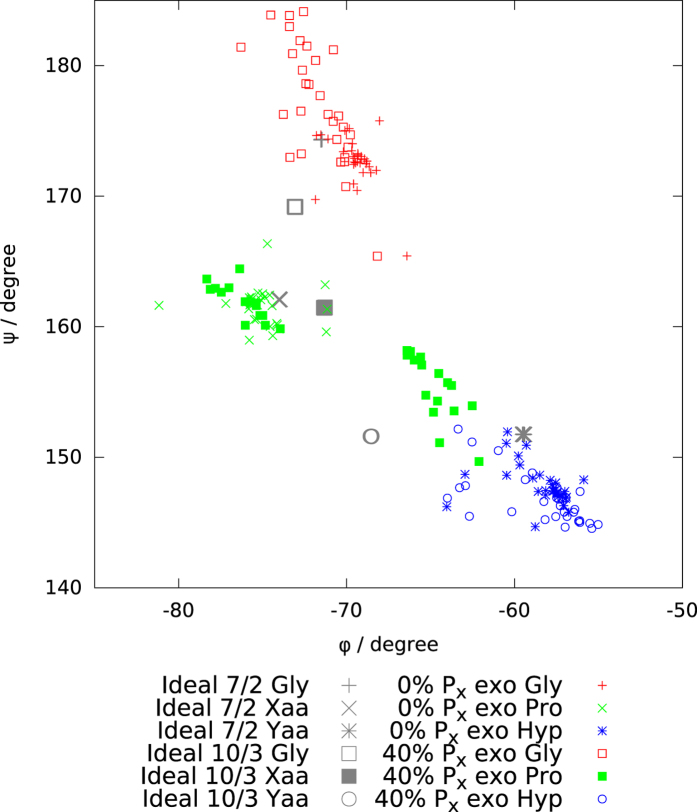
Ramachandran plots of the backbone dihedral angles for idealised 7/2 (imino-rich) and 10/3 (amino-rich) triple helices^38^ and our calculated structures of (POG)_12_ with 0% and 40% exo pucker.

Figure 6 shows Ramachandran plots for all glycine, proline and hydroxyproline residues in these structural models. Whilst the ground state structure dihedral angles cluster around those for the ideal 7/2 helix (ideal 7/2 helical twist is 54°), those for the excited state structure relevant at room temperature correspond to a tighter helical structure, with a helical twist around 59.4°. Interestingly, the P_X_ rings in this excited state structure have backbone dihedral angles fairly evenly distributed about those for an ideal 10/3 helix, rather than 7/2. At temperatures of biological interest, the molecular structure is of course highly dynamic, sampling a wide range of endo:exo ratios and with different rings at any one time adopting endo and exo conformations, and so the Ramachandran plot will be similarly varying with time, but nevertheless, this analysis serves to show how much structural distortion away from ideal 7/2 symmetry can be present at biologically-relevant temperatures, perhaps towards time-averaged P_X_ molecular geometries that are more consistent with those expected for a 10/3 helix than 7/2.

## Discussion

Proline and hydroxyproline rings in both model collagen peptides and native collagens are known to flip between endo and exo conformations on the nanosecond timescale in the solid state. The proline ring conformation is known to be coupled to the peptide backbone ϕ dihedral angle^21^, so that flips between different proline ring conformations can be expected to lead to fluctuating proline dihedral angles. The very rapid timescale of the ring flipping means that for the vast majority of biological interactions, only the time-averaged structure is important, which in turn is simply determined by the relative time-averaged populations of the various conformational states visited by the imino rings, which in this work were directly measured by ssNMR.

By studying model collagen peptides containing GPP and GPO triplets, we have shown that proline has a frustrated structure in the Xaa position of a Gly-Xaa-Yaa triplet in a model collagen peptide when followed by hydroxyproline in the Yaa position and facing hydroxyproline in a neighbouring chain, i.e. for these proline rings, there is no strongly preferred potential energy minimum for any ring conformation. The “fixing” of O_Y_ in the exo conformation with its concomitant backbone dihedral angles, along with those in neighbouring chains, seems to be the factor that confers this metastable structural state on the preceding P_X_. The result is significant structural flexibility at P_X_, with the proline ϕ dihedral angle fluctuating through around 10° as the proline ring moves between endo and exo conformational states according to our calculations. Such flexibility may have biological relevance; XRD studies of proteins binding to collagen-like peptides suggest that the binding produces local “kinks” in the backbone of the peptide chain bound to the protein^2^,^3^,^4^, and local changes in the P_X_ endo:exo ratio towards exo appear to accompany this effect. The broad range of endo:exo ratios for P_X_ rings in GPO triplets predicted to exist at any given temperature implies that P_X_ rings in GPO triplets dynamically sample both high and low endo:exo ratios, perhaps facilitating protein binding or fine-tuning the recognition segment to a particular binding affinity. It is interesting that the dominant equilibrium P_X_ endo:exo ratio at biologically-relevant temperatures is close to 50:50. In pure statistical terms, this represents the most disordered state possible for the ring conformation, and thus a natural “sink” for the triple helical-structure to return to, for example, when binding proteins dissociate.

## Materials and Methods

### Peptide Synthesis and Purification

Peptides were synthesised (0.1 mmol scale) as C-terminal amides on a TentaGel R RAM resin (loading of 0.19 mmol/g, Rapp Polymere) following the standard Fmoc-based solid-phase peptide synthesis strategy on a microwave-assisted automated peptide synthesiser (Liberty^TM^, CEM), and purified by reverse phase HPLC as detailed in Supplementary Information, Section S1.

Pure peptides were characterised by matrix-assisted laser desorption and ionization-time of flight (MALDI-TOF) mass spectrometry, then lyophilised.

### Solid-state NMR

All ssNMR spectra were recorded on a 9.4 Tesla Bruker Avance I NMR spectrometer, operating at frequencies of 400 MHz (^1^H) and 100 MHz (^13^C). Magic angle spinning (MAS) frequency was 10 kHz for all experiments.

Labelled peptides were packed into kevlar inserts (Bruker) which in turn fit into standard zirconia 4 mm rotors (Bruker).

^13^C and ^15^N cross polarization (^13^C CP) experiments: The standard CP sequence in the Bruker pulse program library was used with ^1^H 90° pulse length 2.5 μs, contact time 2.5 ms; during the contact pulse a ramped pulse was applied on ^1^H with average spin lock field of 70 kHz. During acquisition, SPINAL64 decoupling at field strength 100 kHz was applied on ^1^H. Chemical shifts were referenced to external glycine (α polymorph), using the methylene signal at 43.1 ppm relative to TMS (^13^C).

Variable temperature experiment: using a Bruker Variable Temperature Unit, the temperature of the sample was varied from 242 K to 342 K in steps of 5 K. At each temperature, the sample was allowed to equilibrate for up to 10 minutes before a standard ^13^C CP-MAS spectrum was recorded.

### Calculating the Potential Energy Landscape for Puckered States

The GMIN, OPTIM and PATHSAMPLE energy landscape exploration software employing the AMBER9 force field with implicit solvent (igb = 2) and standard amino acid parameters (ff99SB) was used. The non-standard hydroxyproline parameters were derived from ab initio quantum calculations^37^. The initial co-ordinates and topology for (POG)_12_ were generated from PDB 1V7H^9^ and modified to give (PPG)_12_. Full details can be found in Section S2, Supplementary Information.

### Differential Scanning Calorimetry (DSC)

DSC was performed using a Mettler Toledo DSC 822 on the solid, as-prepared peptides with 80 cm^3^/min N_2_ and a heating rate of 5 °C/min from 20 °C to 70 °C while the power required to maintain this rate was monitored.

## Additional Information

**How to cite this article**: Chow, W. Y. *et al.* Hydroxyproline Ring Pucker Causes Frustration of Helix Parameters in the Collagen Triple Helix. *Sci. Rep.* **5**, 12556; doi: 10.1038/srep12556 (2015).

## Supplementary Material

Supplementary Information

## Figures and Tables

**Table 1:** Proline ^13^C chemical shifts showing the variation between Xaa and Yaa positions, preferred pucker, and for endo and exo proline rings in poly(Pro)^26^.

Code	C’	C_α_	C_β_	C_γ_	C_δ_	Pucker	Position	Sequence
**GPO-GX**	171.6	58.4	28.0	25.3	46.9	endo	Xaa	(GPO)_5_G*P*O(GPO)_5_†
**GPP11-Y**	174.4	58.9	29.6	25.7	47.5	exo	Yaa	(GPP)_5_GPP*(GPP)_5_‡
**GPP-YG**	174.4	58.8	29.5	25.7	47.6	exo	Yaa	(GPO)_5_GPP*G*PO(GPO)_4_+
**GPP-GX**	171.3	58.2	28.1	24.4, 25.0	46.7	endo	Xaa	(GPO)_5_G*P*P(GPO)_5_+
Form “A”^26^	170.8	58.8	28.6	23.8	47.7	endo	–	poly(L-proline) II, 11–44°C
Form “B”^26^				25.5		exo	–	poly(L-proline) II, 11–44°C

A full list of assignments is presented in Table S1, Supplementary Information. PDB structure entries are given as footnotes below the table for crystalline analogues of the peptides where they exist.

^†^PDB 3B0S^20^.

^‡^PDB 1K6F^7^ and PDB 3AH9^30^.

^⊕^No PDB entry matches exactly, PDB 1K6F and 3AH9 were used as references.

**Table 2:** Changes in backbone dihedral angles upon flipping from ground state to the opposite puckered state, given as averages and standard deviations across each proline class.

Proline class*	**Δϕ**		**Δψ**	
	**Avg**	**SD**	**Avg**	**SD**
(PPG)_12_ P_X_	−11.08	0.18	6.90	0.32
(PPG)_12_ P_Y_	−3.83	0.22	0.28	0.33
(POG)_12_ P_X_	−11.06	0.36	5.16	0.42
(POG)_12_ O_Y_†	−5.85	1.97	1.94	0.85

All values are in degrees.

^*^For P_Y_ or O_Y_ the change in pucker is overwhelmingly exo to endo. For P_X_, the change in pucker can be either way depending on whether the ground state is endo or exo.

^†^Two distinct populations of changes in backbone dihedral angles were observed for (POG)_12_ O_Y_, with Δϕ centred at −4.6° and −9.5°.
