# Supplementary material for: Hydroxyproline Ring Pucker Causes Frustration of Helix Parameters in the Collagen Triple Helix
Source: Sci Rep. 2015 Jul 29;5:12556. doi: 10.1038/srep12556 (PMC4518226; doi:10.1038/srep12556)

Supporting Information for

Hydroxyproline Ring Pucker Causes Frustration of Helix Parameters in the Collagen Triple Helix

W. Ying Chow,[1,*] Dominique Bihan,[2,*] Chris J. Forman,[1] David A. Slatter,[3] David G. Reid,[1] David J. Wales,[1,†] Richard W. Farndale,[2,†] and Melinda J. Duer[1,†]

1 Department of Chemistry, University of Cambridge, Lensfield Road, Cambridge CB2 1EW, UK

2 Department of Biochemistry, University of Cambridge, Downing Site, Cambridge CB2 1QW, UK

3 Institute of Infection and Immunity, School of Medicine, Cardiff University, Cardiff CF14 4XN, UK

∗ Equal contributions.

† Corresponding authors.

Section S1: Peptide synthesis

Peptides were synthesized (0.1 mmol scale) as C-terminal amides on a TentaGel R RAM resin (loading of 0.19 mmol/g, Rapp Polymere) following the standard Fmoc-based solid-phase peptide synthesis strategy on a microwave-assisted automated peptide synthesizer (Liberty™, CEM).

Fmoc deprotection (20% (v/v) piperidine in dimethyl- formamide (DMF)) was performed in two stages with an initial deprotection of 30 s followed by 3 min at 70 °C (52 W microwave power). Coupling of Fmoc-amino acids (0.5 mmol, 0.2 M in DMF) was carried out using HCTU (2-(6-chloro-1-H-benzotriazole-1-yl)-1,1,3,3-tetramethyluronium hexafluorophosphate) (0.5 mmol, 0.5 M in DMF) and *N,N*-diisopropylethylamine (10 mmol, 2M in *N*-methyl-pyrrolidone). All couplings were performed for 5 min at 70 °C (25 W microwave power) except for the following amino acids: first Fmoc-amino acid coupled to the resin was double coupled; Fmoc-[U-^{13}C, ^{15}N]-labeled amino acids (Cambridge Isotope Laboratories) were coupled for 10 min. Cleavage of the peptides from the resin and simultaneous side-chain deprotection was achieved by treatment of the peptide-resin with a trifluoroacetic acid, water and triisopropylsilane mixture (95:2.5:2.5 v/v, 10 mL) for 3 h. The resin was filtered and the filtrate concentrated under reduced pressure to ca. 1 mL volume, after which the crude peptides were precipitated with ice-cold ether. The filtered crude peptides were ether-washed (twice), dissolved in 5% acetonitrile in water containing 0.1% trifluoroacetic acid and then lyophilized. Crude peptides were purified by reverse phase high performance liquid chromatography (PerkinElmer Life Sciences LC200) on an ACE 10 Phenyl-300 column (250x21.2 mm, Hichrom Ltd) using a linear gradient of acetonitrile in water containing 0.1% trifluoroacetic acid. The pure peptides were characterized by matrix-assisted laser desorption/ionization-time of flight (MALDI-TOF) mass spectrometry, and then lyophilized.s

Mass spectra of the peptides used in this study are shown on the subsequent pages.

**GPO-GX**	$(GPO)_5$**G*P***O$(GPO)_5$	Page 2	Amount: 20.0 mg
**GPP11-Y**	$(GPP)_5$GP**P***$(GPP)_5$	Page 3	Amount: 10.3 mg
**GPP-YG**	$(GPO)_5$GP**P*G***PO$(GPO)_4$	Page 4	Amount: 13.0 mg
**GPP-GX**	$(GPO)_5$**G*P***P$(GPO)_5$	Page 5	Amount: 14.1 mg

GPO-GX (GPO)$_5$**G*P***O(GPO)$_5$

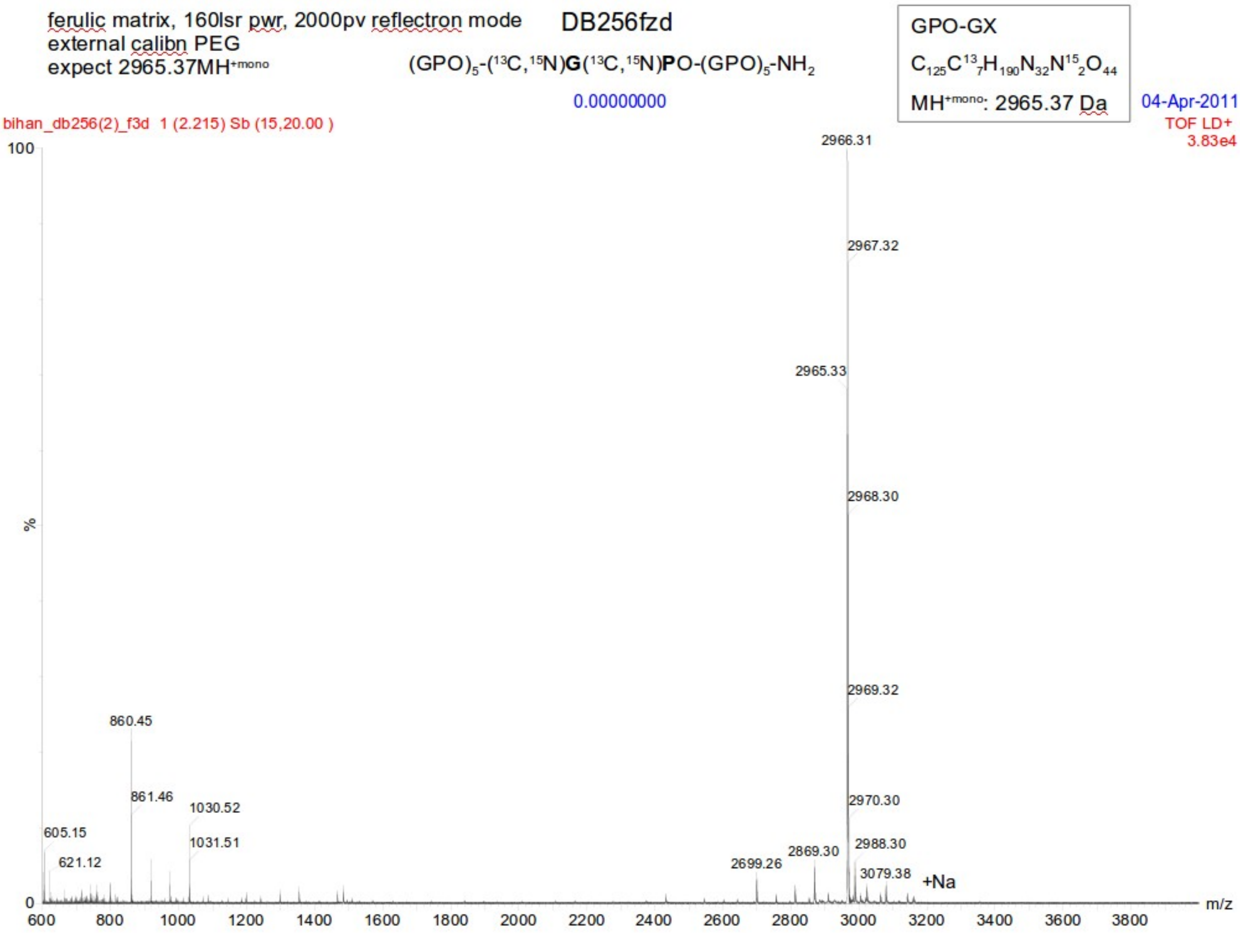

GPP11-Y (GPP)$_5$GP**P***(GPP)$_5$

This sample was combined from three higher purity fractions.

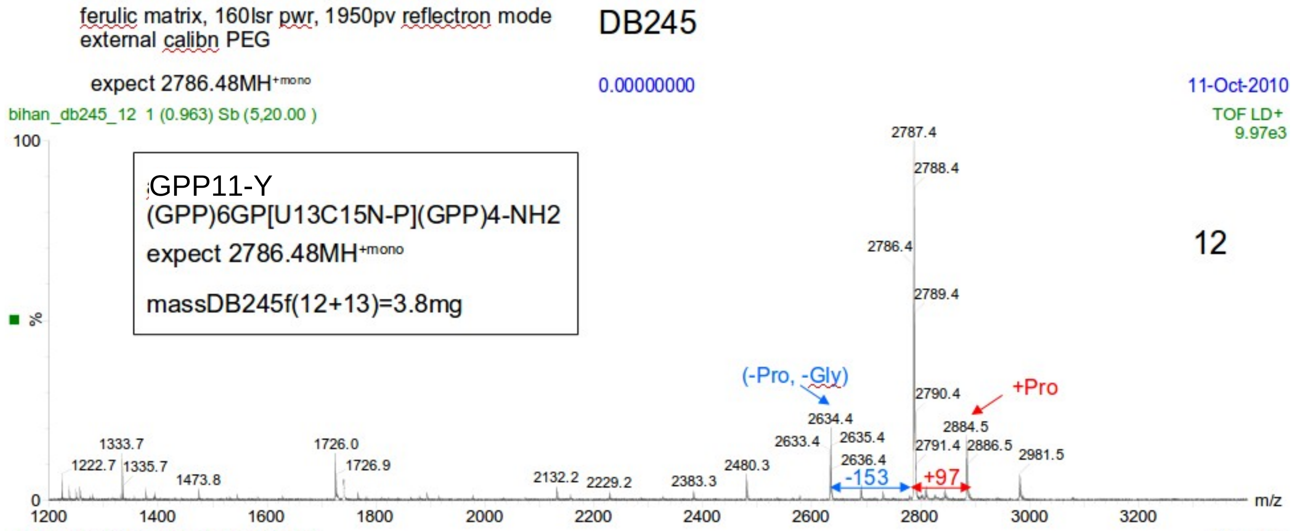

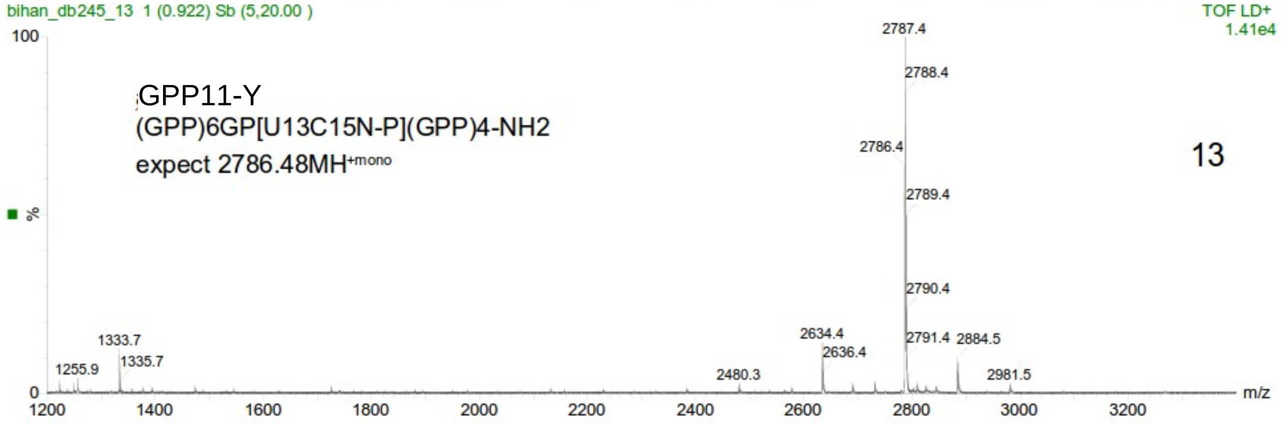

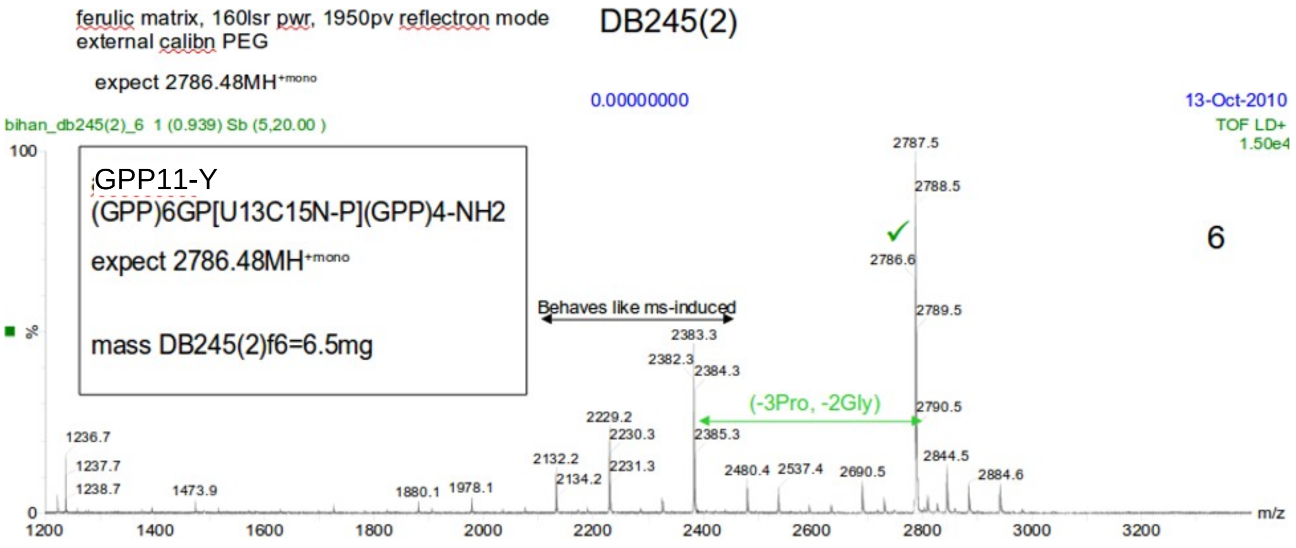

GPP-YG (GPO)$_5$GP**P*G***PO(GPO)$_4$

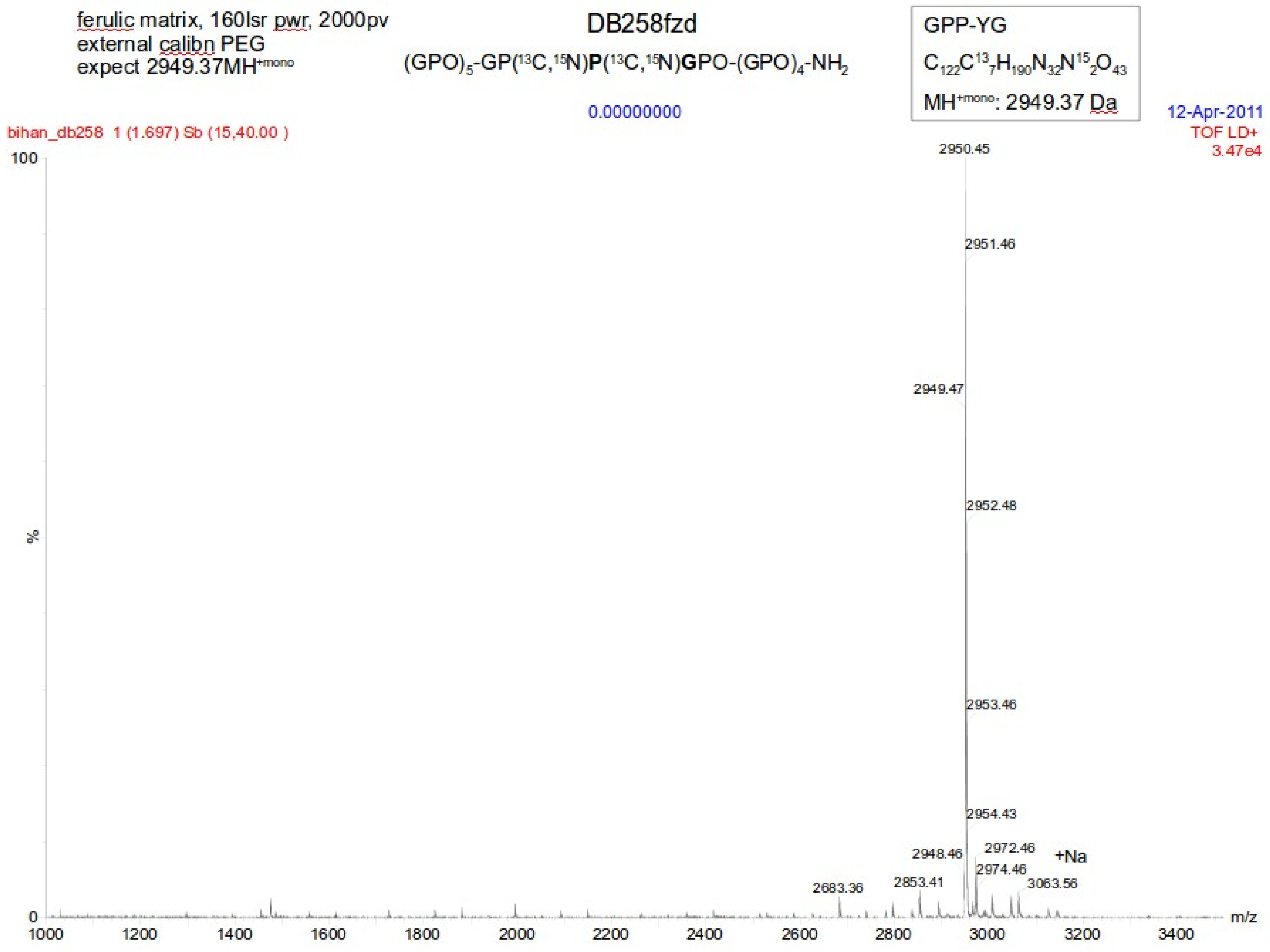

GPP-GX $(GPO)_5$**G*P***P$(GPO)_5$

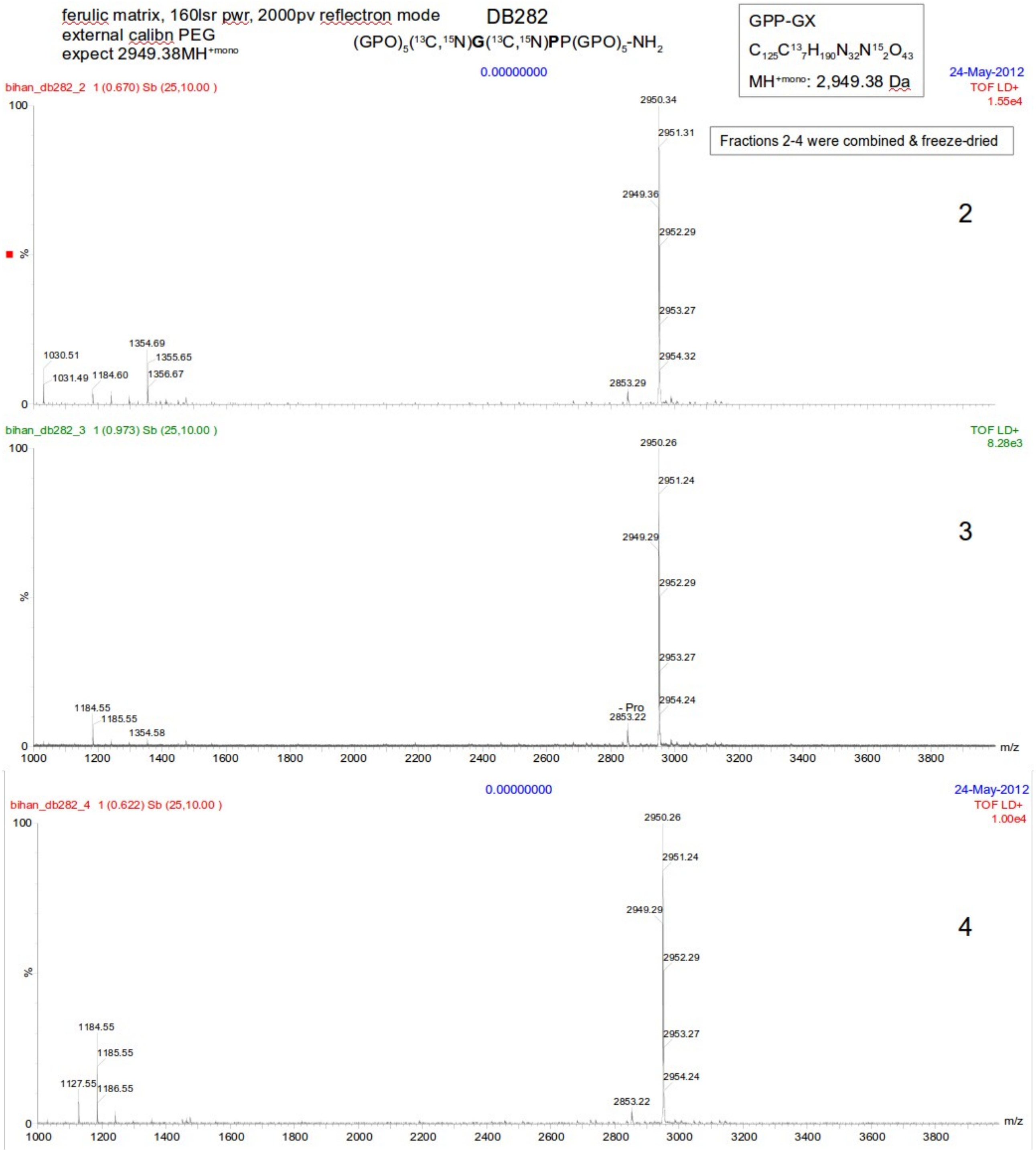

Section S2: Further Details on Computational Methods

Molecular Modeling of $(PPG)_{12}$ and $(POG)_{12}$. The initial atomic coordinates and structural information for $(POG)_{12}$ was generated from $(POG)_{10}$ (PDB 1V7H), an experimentally derived model peptide structure possessing 7/2 symmetry. A $(POG)_{2}$P sub-segment of $(POG)_{10}$ was extracted and attached at the N terminus by overlapping and aligning the terminal proline residues of the two sub-segments. After discarding one of the overlapping prolines the resulting $(POG)_{12}$ structure was terminated by ACE and NME groups at the N and the C termini of each chain respectively. $(PPG)_{12}$ was easily generated from $(POG)_{12}$. The additional eight residues per chain (including NME and ACE) were added to mitigate end effects, facilitating direct comparison of the central $(POG)_{10}$ and $(PPG)_{10}$ sequences with longer native collagen systems. The entire sequence is numbered from residue 1 to 114, consisting of three chains (A: 1 to 38, B: 39 to 76, C: 77 to 114). Initial structures were generated for probing the potential energy landscape.

The re-organization strategy combined short runs of molecular dynamics at a variety of temperatures (300 K to 700 K), small pseudo-random changes to the Cartesian coordinates, and a bespoke proline pucker flipping move that rotates the $C\gamma$ carbon group about the C_β-C_δ axis by arbitrary amounts. The pucker flipping move artificially forces the tip of the proline ring to one side or the other of the endo--exo transition point and subsequent relaxations allow the proline ring to adopt one of two minima – either endo or exo – separated by the transition state. Ten thousand basin-hopping steps were performed for each system. This procedure generated 4S stereo-isomers of the OH group, which were discarded, since these are not present in natural collagen.

Locating the Potential Energy Landscape Global Minimum. The lowest energy minimum after ten thousand steps was employed as the putative global minimum in a simple algorithm that systematically flipped the pucker state of each proline ring independently. If a ring flip generated a minimum with a lower energy than the putative ground state then the original was replaced and the algorithm restarted. This searching process was repeated until all 72 proline rings (including those in the end caps) could be flipped from the same starting minimum without generating a lower minimum. The resulting common ground state was taken as the global minimum.

Calculating the Energy Landscape for Puckered States. Starting with the global minimum, we systematically (and independently) flipped each of the proline rings to generate up to 72 separate higher energy minima that possessed a single flipped proline ring. Interestingly $(POG)_{12}$ minima corresponding to the excited pucker states of residues O_Y27, P_X99 and O_Y100 could not be found, and probably do not exist.

Starting with each of the higher energy minima whose single flipped residue was in the central 30 residues of each chain (A:5-34, B:43-72 and C:81-110), we systematically flipped each of the other rings, to generate minima with two flipped rings.

Each pair of minima in the resulting set can be connected by a pathway that consists of alternating transition states and minima. The PATHSAMPLE program, which drives OPTIM, can be used to find such pathways through the landscape. The software builds a relational database as it goes, adding new minima as they arise and storing information about which transition states are directly connected with which adjacent minima In most of the cases that we explored, the global minimum could be successfully connected to each of the single flip minima by a pathway consisting of a single transition state. Similarly, most of the single flip minima could be connected to double flip minima by a single transition state, although there are multiple routes from the ground-state to the second flip minima.

Accurate characterization of the transition states between each pair of minima is critical for meaningful subsequent thermodynamic and kinetic analysis of the energy landscape, and there may be more than one path between each pair. The OPTIM program efficiently determines these minimal routes through the landscape using well defined algorithms.

In a few cases there were intermediate transition states involving the rotation of the OH group but, compared to the energy barrier of changing the pucker state, such transitions are very fast and states on either side of an OH rotation transition state were treated as the same pucker state. In total for $(POG)_{12}$ 6481 transition states, 2994 minima and 1712 distinct ring pucker states consisting of either 0, 1 or 2 flipped rings were found for $(POG)_{12,}$ whereas we discovered for $(PPG)_{12}$ 3609 transition states connecting 2203 minima with 1839 distinct ring pucker states. All these minima and transition states are stored in a database along with connectivity information, and a variety of auxiliary information, such as point group order and vibrational frequencies from harmonic normal mode analysis.

Each minimum in the database was analyzed to determine which rings had flipped from the ground state and to classify them according to Xaa and Yaa positions in the collagen GXY sequence. The χ_i, φ and ϕ dihedral angles were measured for every residue along every minimum structure, where the index i (taking values of 1-5) represents the dihedral angles around the proline ring. When the C_α-C_β-C_γ-C_δ dihedral, i.e. χ_2, is less than 0, the ring is considered to be in the endo state. This is a slightly broader definition of endo (and exo) compared to some previous work[refs: Ho et al, Protein Sci, 2005; Ramachandran et al Biochim Biophys Acta 1970], but the difference in endo:exo proportion as calculated using our definition is less than 10%. In addition all the hydrogen bond lengths were computed between the relevant amide groups in adjacent chains.

Based on the harmonic vibrational densities of state and the size of the intervening barrier, the forward and backward transition rates between any two minima in the database can be calculated using transition state theory for any given temperature. The equilibrium occupation probabilities can be found with the same harmonic approximation allowing an estimate of the relative probability of finding each proline ring in its endo or exo state on either side of the intervening transition state.

Table S1: ^{13}C and ^{15}N Assignments of Peptides in this Study. ^{15}N chemical shifts were referenced to external glycine (α polymorph) using the glycine amino signal at 32.3 ppm relative to ammonia.

Code	Sequence	Glycine		Proline				
		C'	Cα	C'	Cα	Cβ	Cγ	Cδ
**GPO-GX**	$(GPO)_5$**G*P***O$(GPO)_5$–NH_2	167.8	42.7	171.6	58.4	28.0	25.3	46.9
**allGPP-Y**	$(GPP)_5$GP**P***$(GPP)_5$–NH_2	-	-	174.7	58.9	29.6	25.7	47.5
**GPP-YG**	$(GPO)_5$GP**P*G***PO$(GPO)_4$–NH_2	167.8	42.7	174.4	58.8	29.5	25.7	47.6
**GPP-GX**	$(GPO)_5$**G*P***P$(GPO)_5$–NH_2	167.9	42.6	171.3	58.2	28.1	24.4, 25.0	46.7
**GPG1**	$(GPO)_5$**G*P***$(GPO)_5$–NH_2	168.7	42.3	174.3	59.0	29.9	25.0	47.0
**GPG2**	$(GPO)_5$G**P*G***PO$(GPO)_4$–NH_2	167.9	41.7	174.2	59.0	29.9	24.9	46.9

Code	Sequence	Glycine	Proline
		N	N
**GPO-GX**	$(GPO)_5$**G*P***O$(GPO)_5$–NH_2	104.1	128.4
**allGPP-Y**	$(GPP)_5$GP**P***$(GPP)_5$–NH_2	104.1	129.5
**GPP-YG**	$(GPO)_5$GP**P*G***PO$(GPO)_4$–NH_2	103.3	130.1
**GPP-GX**	$(GPO)_5$**G*P***P$(GPO)_5$–NH_2	104.4	128.8
**GPG1**	$(GPO)_5$**G*P***$(GPO)_5$–NH_2	107.5	130.6
**GPG2**	$(GPO)_5$G**P*G***PO$(GPO)_4$–NH_2	105.1	131.2

Figure S1: ^{13}C CP-MAS ssNMR spectra of the triple-helical peptides **GPP-GX** as a function of temperature.

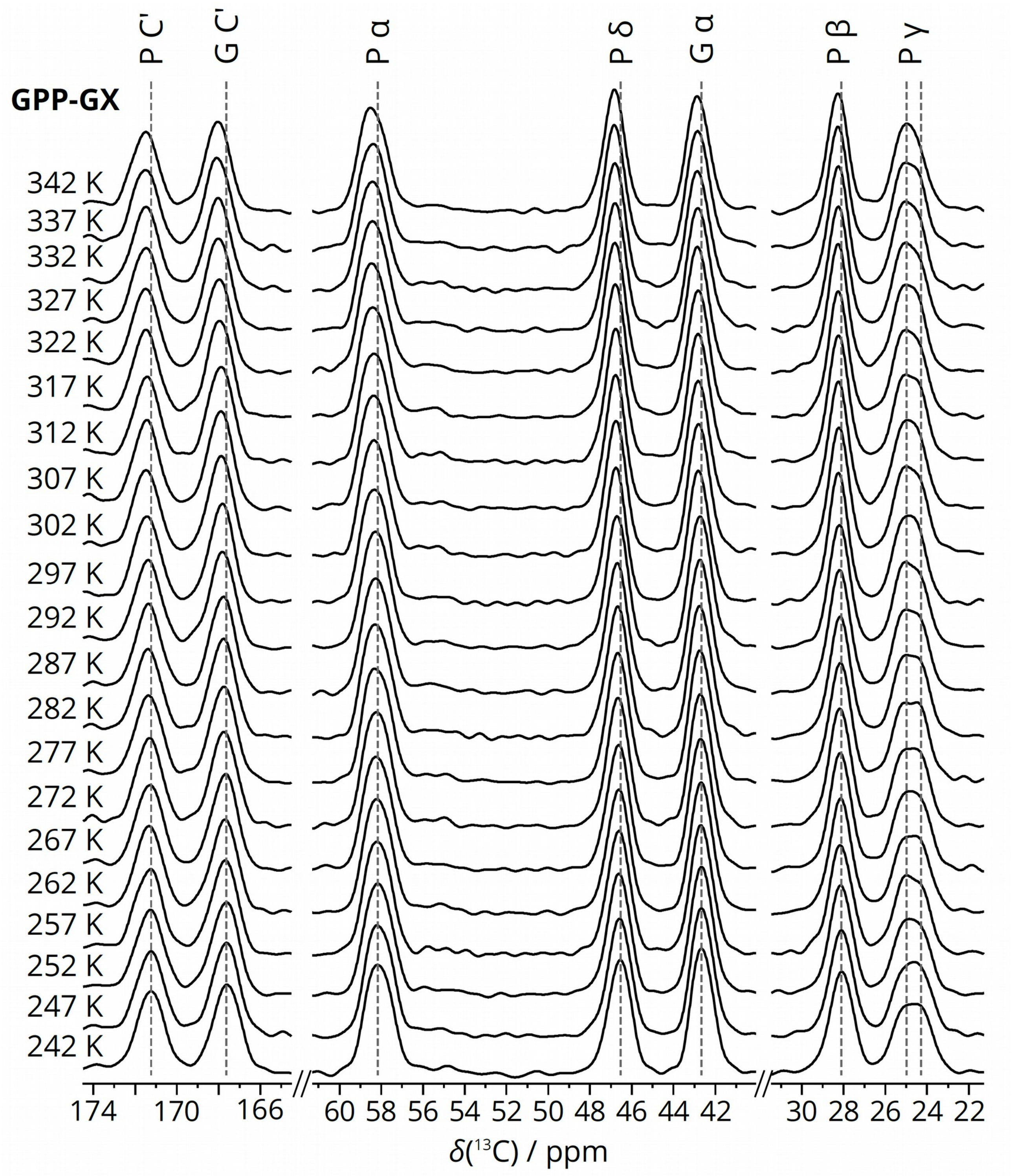

Figure S2: ^{13}C CP-MAS ssNMR spectra of the triple-helical peptides **GPO-GX** as a function of temperature.

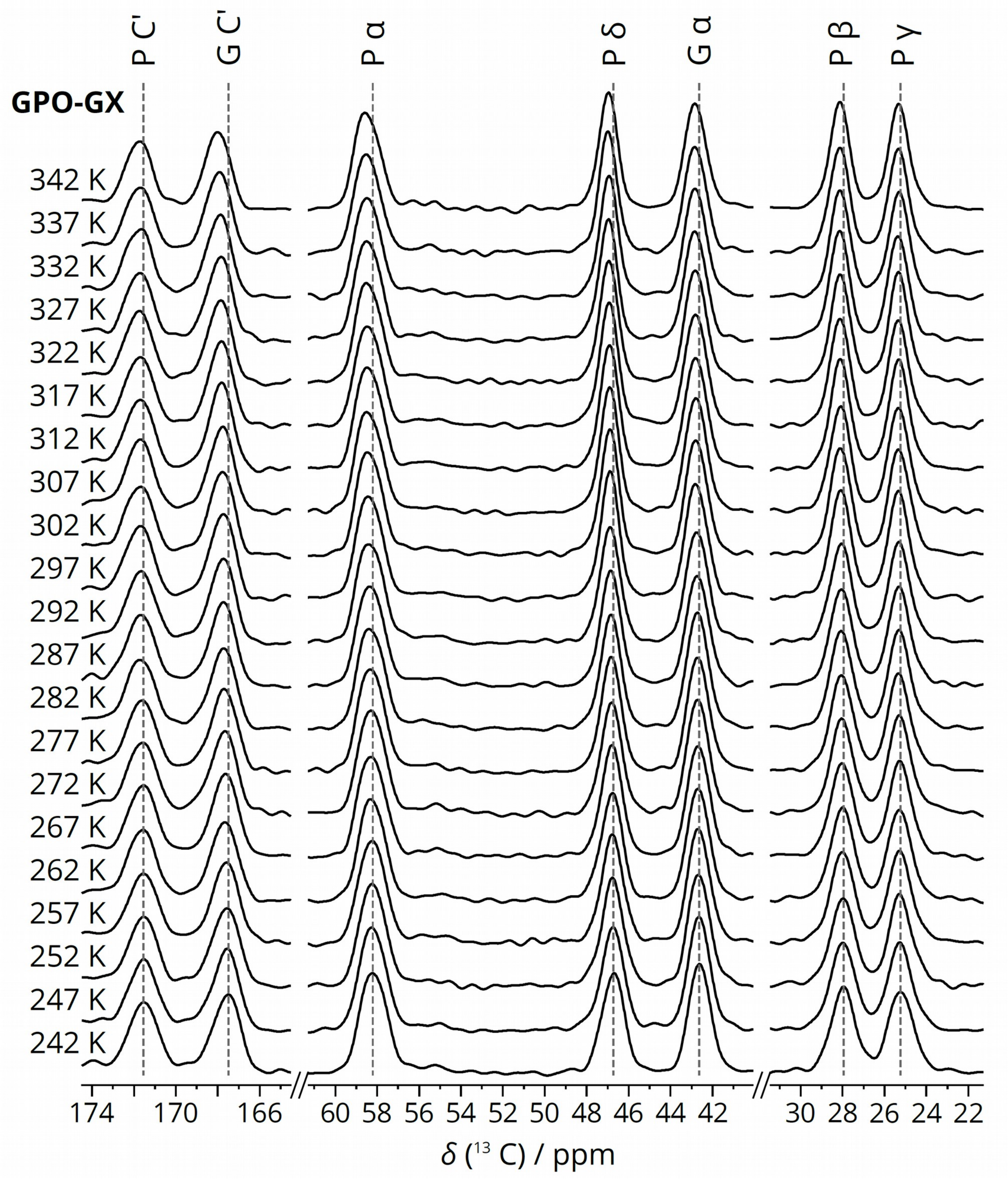

Table S2: ^{13}C peak maximum chemical shifts for **GPO-GX** as a function of temperature**.**

T/ K	ProCO	GlyCO	ProCα	ProCδ	GlyCα	ProCβ	ProCγ
242	171.53	167.49	58.23	46.69	42.62	27.95	25.24
247	171.53	167.55	58.27	46.74	42.65	27.99	25.27
252	171.53	167.56	58.24	46.77	42.67	27.97	25.29
257	171.55	167.58	58.24	46.78	42.68	28.01	25.28
262	171.56	167.66	58.32	46.77	42.69	27.99	25.29
267	171.54	167.63	58.30	46.79	42.69	28.03	25.27
270	171.63	167.68	58.40	46.81	42.71	28.05	25.32
272	171.58	167.65	58.30	46.81	42.70	28.05	25.29
277	171.61	167.70	58.33	46.83	42.71	28.06	25.33
282	171.72	167.72	58.40	46.84	42.72	28.07	25.31
287	171.67	167.73	58.36	46.85	42.73	28.05	25.30
292	171.66	167.72	58.42	46.89	42.75	28.04	25.31
292	171.73	167.76	58.44	46.85	42.77	28.07	25.34
297	171.68	167.73	58.44	46.89	42.82	28.08	25.33
297	171.67	167.85	58.46	46.91	42.78	28.08	25.34
302	171.67	167.77	58.47	46.89	42.80	28.11	25.34
307	171.68	167.76	58.51	46.90	42.78	28.08	25.34
310	171.72	167.88	58.47	46.90	42.79	28.10	25.31
312	171.70	167.82	58.48	46.91	42.80	28.11	25.32
317	171.69	167.82	58.48	46.93	42.81	28.12	25.34
322	171.74	167.85	58.52	46.94	42.82	28.12	25.33
327	171.71	167.84	58.51	46.96	42.85	28.14	25.35
332	171.64	167.87	58.49	46.93	42.83	28.13	25.30
337	171.69	167.91	58.53	46.99	42.85	28.12	25.33
342	171.72	168.01	58.59	46.96	42.84	28.13	25.32

Change of around 0.1~ppm at the bulk water melting or freezing transition around 292-297 K.

Table S3: ^{13}C peak maximum chemical shifts for **GPP-GX** as a function of temperature**.**

T/ K	ProCO	GlyCO	ProCα	ProCδ	GlyCα	ProCβ	ProCγ
242	171.24	167.61	58.17	46.55	42.66	28.09	24.56
247	171.24	167.61	58.20	46.59	42.66	28.10	24.62
252	171.26	167.62	58.16	46.62	42.66	28.14	24.85
257	171.25	167.68	58.21	46.61	42.69	28.17	24.98
262	171.33	167.69	58.22	46.61	42.68	28.14	24.58
267	171.28	167.66	58.25	46.62	42.69	28.16	24.81
270	171.37	167.73	58.32	46.65	42.75	28.18	24.58
272	171.31	167.75	58.22	46.66	42.71	28.18	24.65
277	171.36	167.74	58.32	46.67	42.74	28.15	24.49
282	171.38	167.76	58.29	46.66	42.76	28.17	24.98
287	171.36	167.76	58.27	46.69	42.73	28.19	25.01
292	171.37	167.82	58.27	46.71	42.73	28.20	25.04
292	171.44	167.82	58.30	46.71	42.80	28.19	24.84
297	171.44	167.82	58.33	46.76	42.81	28.25	24.86
297	171.43	167.87	58.31	46.75	42.78	28.24	25.03
297	171.43	167.87	58.33	46.73	42.78	28.22	25.05
297	171.44	167.86	58.34	46.74	42.78	28.23	24.94
302	171.48	167.88	58.35	46.75	42.80	28.22	25.00
307	171.44	167.89	58.36	46.77	42.82	28.25	24.94
310	171.47	167.96	58.37	46.78	42.79	28.22	24.96
312	171.43	167.88	58.34	46.77	42.82	28.24	25.03
317	171.48	167.96	58.41	46.79	42.81	28.26	25.03
322	171.49	167.97	58.46	46.81	42.85	28.26	25.01
327	171.47	168.02	58.42	46.80	42.84	28.25	25.03
332	171.48	168.03	58.42	46.81	42.86	28.25	25.03
337	171.50	168.09	58.38	46.83	42.84	28.27	25.03
342	171.49	168.03	58.52	46.83	42.87	28.27	24.97

Pro Cγ in this table only includes the more intense signal.

Change of around 0.1~ppm at the bulk water melting or freezing transition around 297 K.

Table S4: ^{13}C peak maximum chemical shifts for **GPP-YG** as a function of temperature..

T/ K	ProCO	GlyCO	ProCα	ProCδ	GlyCα	ProCβ	ProCγ
242	173.90	167.69	58.65	47.43	42.43	29.26	25.71
247	174.10	167.65	58.68	47.43	42.44	29.30	25.75
252	174.28	167.71	58.68	47.42	42.41	29.34	25.73
257	174.38	167.63	58.70	47.45	42.46	29.38	25.74
262	174.37	167.72	58.70	47.45	42.45	29.37	25.68
267	174.37	167.80	58.73	47.47	42.50	29.42	25.69
270	174.12	167.72	58.73	47.52	42.50	29.45	25.74
272	174.16	167.78	58.73	47.53	42.46	29.39	25.72
277	174.27	167.77	58.77	47.51	42.51	29.43	25.73
282	174.23	167.83	58.73	47.52	42.52	29.44	25.73
287	174.21	167.85	58.79	47.54	42.53	29.44	25.76
292	174.24	167.79	58.82	47.55	42.53	29.50	25.79
292	174.30	167.77	58.81	47.54	42.54	29.42	25.73
297	174.14	167.92	58.82	47.58	42.61	29.48	25.79
297	174.11	167.91	58.83	47.56	42.55	29.46	25.83
302	174.24	167.86	58.88	47.61	42.57	29.56	25.79
307	174.28	167.91	58.86	47.58	42.59	29.55	25.80
310	174.24	167.93	58.87	47.63	42.59	29.57	25.78
312	174.34	167.94	58.88	47.59	42.61	29.54	25.76
317	174.40	167.97	58.89	47.63	42.64	29.53	25.75
322	174.39	167.92	58.91	47.64	42.63	29.53	25.77
327	174.47	167.97	58.92	47.64	42.62	29.55	25.84
332	174.31	167.98	58.93	47.64	42.66	29.53	25.76
337	174.31	167.98	58.93	47.64	42.66	29.53	25.76
342	174.42	168.03	58.95	47.67	42.66	29.60	25.80

Figure S3: Differential scanning calorimetry (DSC) traces for **GPP-GX** and **GPO-GX**.

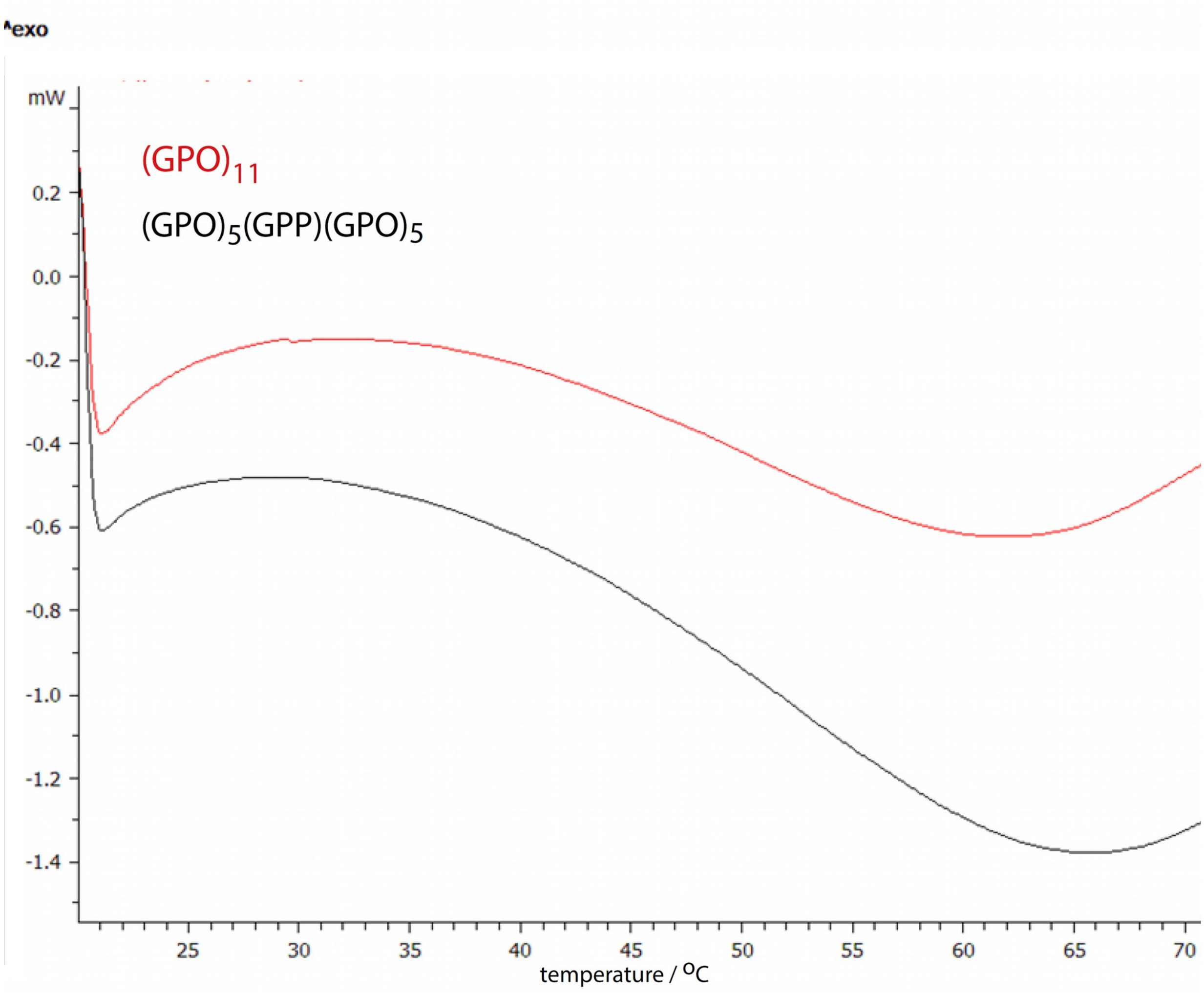

Section S3: Further analysis of energy landscape modelling

The energy changes between ring conformations are seen most clearly in the so-called disconnectivity graphs shown in Figure S4. In disconnectivity graphs, the multidimensional potential energy landscape resulting from calculations is depicted graphically to highlight pathways and barriers involved in the interconversion of related configurations, in order to provide insight into structure, dynamics and thermodynamics of the system. In this study, the potential or free energy of each minimum determines its vertical position, and the minima are colored according to the chain position, i.e. X or Y in a GXY triplet, of the flipped rings in the structure. A carefully chosen representation has been used in these graphs to highlight the distribution of energies for the excited state structures with zero, one or two flipped rings.

The global minimum is represented as the black group in the center at the lowest point of both graphs. The disconnectivity diagram for $(POG)_{12}$ exhibits three main energy bands, which correspond to the number of flipped hydroxyprolines. From the expanded diagram for $(POG)_{12}$ (Fig S4), the lowest energy band can be examined. In this expanded view, the black sub-band is the ground state with no flipped rings; the red sub-band has one flipped P_X ring; and the blue sub-band has two flipped P_X rings. Fig S4 shows that flipping two P_X rings rather than one does not significantly alter the energy within the band---only by about 0.3 kcal/mol---suggesting that P_X endo and exo conformations have similar thermodynamic stabilities in $(POG)_{12}$.

States with a single flipped hydroxyproline correspond to the combined green (single hydroxyproline ring flipped) and magenta (one hydroxyproline and one proline (X) flipped) bands. Again, flipping the P_X position has comparatively little effect on the energy within this band of ring conformational states. Those states with two flipped hydroxyproline rings corresponds to the yellow band (no $P_X$rings flipped), which is significantly higher in energy.

The range of energies occupied by the minima in each band is very tight, suggesting that puckering of any two rings that fulfill the grouping criteria (Xaa or Yaa, proline or hydroxyproline) will requires similar energies, and therefore that there is little coupling between rings.

Figure S4: Disconnectivity diagram calculated for $(POG)_{12}$ and $(PPG)_{12}$ for no flips (ground state), one flip and two flips. Colors indicate the classification of the minima according to number of flips (single or double), ring position (Xaa or Yaa), and residue type (proline or hydroxyproline). Note that the three diagrams have different vertical energy scales, indicated by the 1 kcal/mol label.

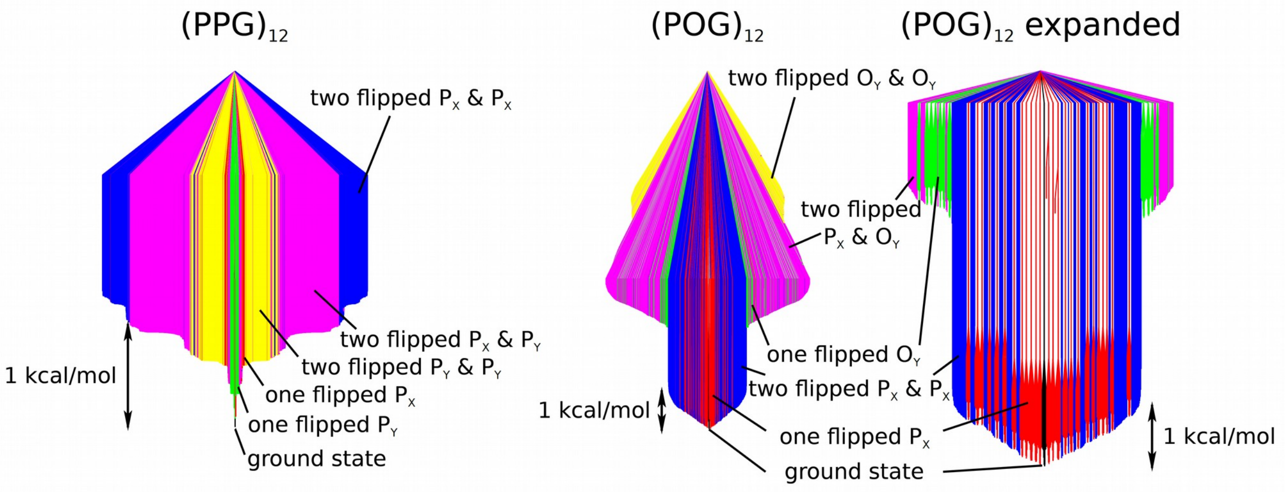

Supplement: Supplementary Information [file srep12556-s1.pdf]
